# Role of epigenetics-microRNA axis in drug resistance of multiple myeloma

**DOI:** 10.1186/s13045-017-0492-1

**Published:** 2017-06-17

**Authors:** Nasrin Rastgoo, Jahangir Abdi, Jian Hou, Hong Chang

**Affiliations:** 1 Division of Molecular and Cellular Biology, Toronto General Research Institute, Toronto, Canada; 2 Department of Laboratory Medicine & Pathobiology, University of Toronto, Toronto, Canada; 3 Department of Hematology, Shanghai Chang Zheng Hospital, Shanghai, China; 4 Department of Laboratory Hematology and Medical Oncology, University Health Network, 200 Elizabeth Street, 11E-413, Toronto, ON M5G 2C4 Canada

**Keywords:** Epigenetics, EZH2, MicroRNA, Myeloma, Drug resistance

## Abstract

Despite administration of novel therapies, multiple myeloma (MM) remains incurable with resistance to drugs leading to relapse in most patients. Thus, it is critical to understand the detailed mechanisms underlying the drug resistance of MM and develop more effective therapeutic strategies. Genetic abnormalities are well known to play a central role in MM pathogenesis and therapy resistance; however, epigenetic aberrations mainly affecting the patterns of DNA methylation/histone modifications of genes (especially tumor suppressors) and miRNAs have also been shown to be involved. Importantly, while epigenetic silencing of miRNAs in MM is well documented, some epigenetic markers are known to be direct targets of miRNAs particularly the recently described “epimiRNAs”. Drugs targeting epigenetic modifiers (e.g., HDACs, EZH2) can sensitize MM-resistant cells to anti-myeloma drugs and reversibility of epigenetic changes makes these drugs promising therapeutic agents. Therefore, combination of miRNA mimics with inhibitors of epigenetic modifiers would be a more potent therapeutic strategy in MM patients in relapse or refractory to treatments. In this review, we will discuss the findings of recent investigations on epigenetics/miRNA regulatory axis in development of drug resistance in MM and highlight possible approaches for therapeutic applications of such interaction.

## Background

Multiple myeloma (MM) is a clonal expansion of plasma cells that is characterized by proliferation of malignant clones producing defective monoclonal immunoglobulins in the bone marrow. Current therapies such as proteasome inhibitors (PIs) and immunomodulatory drugs (IMiDs) have improved the outcome of patients. Nevertheless, not all patients respond well to the drug, and even in responding patients usually relapse occurs. Thus, multidrug resistance is still the major problem for the effective treatment of multiple myeloma with conventional drugs [1, 2]. Researches to decipher the molecular mechanisms underlying drug resistance (DR) of MM are keeping an unstoppable trend with miRNAs and epigenetics leading a fast-growing front. Indeed miRNAs and epigenetic markers have been identified as critical regulators of expression and function of oncogenes/tumor suppressors in cancers including MM [3]. In line with this notion, wealth of evidence supports that epigenetic dysregulations such as aberrant DNA and histone methylation, histone deacetylation and abnormal miRNA expression are involved in the pathogenesis of MM [4–6] (listed in Table 1). This has pushed several studies in recent and past years toward assessment of epigenetic inhibitors in clinical trials of MM [7–9]. Moreover, since the epigenetic alterations are reversible, inhibition of epigenetic changes could have a promising therapeutic potential [10]. Importantly, although aberrant miRNA expression in MM due to epigenetic silencing mechanisms is well documented [11], miRNAs have also been shown to impact epigenetic modifiers in cancers [12–14] highlighting a regulatory circuit between these two regulatory systems. This concept will introduce an exciting venue to DR investigation and therapeutic targeting in MM. However, while miRNAs can play regulatory roles in drug response of MM cells [15], further studies are still required to fully elucidate whether interaction of epigenetic modulations with miRNAs contributes to DR in MM. Here to present a new mechanistic picture based on the most recent findings, we discuss epigenetic abnormalities associated with miRNAs that are involved in DR of MM.

**Table 1:** Aberrant miRNAs involved in pathogenesis or drug resistance of MM

miRNAs	Dysregulation	Outcome	Refs.
miR-21	Upregulation	Inhibition of apoptosis and increase drug resistance	[74]
miR-125b	Upregulation	Reduction of cell death in dexamethasone induced MM (drug resistance)	[75]
miR-15a miR-16	Downregulation	Regulation of MM cell proliferation in vitro and in vivo Downregulated in patients with relapsed/refractory disease (drug resistance)	[76, 77]
miR-221/222	Upregulation	Inhibition of apoptosis and modulation of drug influx-efflux and ABC transporters (drug resistance)	[36]
miR-27a	Downregulation	Downregulated in MM patients with bortezomib-refractory status (drug resistance)	[46]
miR-149	Downregulation	Downregulated in glucocorticoid resistant MM cells by disturbing epigenetic landscape, leading to overexpression of MMP-9 gene which is involved in bone remodeling and tumor invasion in MM	[78]
miR-631	Downregulation	Modulates UbcH10/MDR1 pathway which is associated with the development of BTZ resistance in myeloma cells	[79]
miR-202	Downregulation	Involved on drug resistance of MM cells by targeting JNK/SAPK signaling pathway	[80]
miR-30c	Downregulation	Downregulated as a result of interaction between MM cells and bone marrow stromal cells, which in turn activation of oncogenic Wnt/β-catenin/BCL9 pathway and promote MM cell proliferation, drug resistance and formation of MM cancer stem cells.	[81]
miR-137/197	Downregulation	Modulates MCL-1 which is dysregulated in multiple myeloma cells and overexpression of MCL-1 is associated with relapse and poor survival	[51]
miR-17-92 cluster miR-106~363 cluster	Upregulation	High level is associated with shorter overall survival	[82]
miR-148a & miR-20a	Upregulation	Shorter relapse-free survival	[83]
let-7e, miR-125a-5p, and miR-99b cluster	Upregulation	Overexpression in t(4;14) patients	[84]
miR-140-3p	Downregulation	Altered expression due to the occurrence of several allelic imbalances or loss of heterozygosity in 16q2 region	[84]
*miR-32* and *miR-17*~*92* cluster	Upregulation	Upregulated in MM patients and cell lines but not in MGUS or healthy PCs	[85]
*miR-19a* and *19b*	Upregulation	Inhibition of IL-6 growth signaling	[85]
*miR106b*~*25* cluster, *miR-181a*/*b*, *miR-32*	Upregulation	Targeting of the genes which involved in p53 regulation	[85]
miR-1/miR-133a cluster	Upregulation	Overexpressed in MM patients with t(14;16)	[86]
miR-135b and miR-146a	Downregulation	Downregulated in MM with t(4;14) and targeted the genes which are involved in IL-1 signaling pathway	[86]
miR-214	Downregulation	Positive regulation of P53 and inhibition of DNA replication	[87]
miR-29b	Downregulation	Reduction of apoptosis by upregulation of MCL1	[88]
miR-192, miR-194, miR-215	Downregulation	p53-inducible microRNAs which modulate MDM2 expression regulate IGF pathway and enhance migration of plasma cells into bone marrow	[89]

### Epigenetic dysregulation and DR in MM

Although the molecular mechanisms of DR in MM are not fully understood, epigenetic abnormalities have been suggested to play an important role [16]. In fact the role of DNA methylation, histone modifications, and chromatin remodeling in MM development/progression have been well described [3–6]; however, the mechanistic role of these alterations in DR/relapse of MM has not been fully investigated. Dysregulation of DNA methylation is one of the most studied epigenetic mechanisms in DR of different types of cancers including MM as evidenced by higher frequency of hypermethylation of some tumor suppressor genes, such as CDKN2A and CDKN2B, in relapsed than in newly diagnosed MM patients [17].

In addition, DNA hypermethylation has been detected in some tumor suppressor, cell signaling, and cell adhesion molecule genes in plasma cell leukemia (PCL) cells [18]. Analyzing data from thousands of cancer cell lines and tumors showed that suppressed expression of one or more 19S proteasome subunits caused by DNA methylation led to intrinsic proteasome inhibitor resistance [19]. Furthermore, bone marrow microenvironment-mediated global DNA hypermethylation has been suggested to be involved in DR of MM by upregulating DNA methyl transferases (DNMTs) [20]. Interestingly, it was shown that the oxidative epigenetic agent, RRx-001, inhibited DNMTs and reduced global hypermethylation leading to decrease in viability of MM cells and overcame DR. Of note, microarray screening for genes silenced by DNA methylation revealed an association between gene inactivation by DNA hypermethylation and dexamethasone resistance in MM and treating MM cells with demethylating agent 5-aza-2’-deoxycytidine restored sensitivity to dexamethasone [21]. In addition to DNA methylation, histone modification is also critical in cellular programming and dysregulation of the histone-modifying enzymes is involved in the pathogenesis of MM. Histone deacetylases (HDACs) are dysregulated in MM, and aberrant overexpression of class I HDACs is correlated with reduced overall survival of patients with MM [22]. HDAC inhibitors, including panobinostat and vorinostat, have been evaluated in the treatment of MM and recently approved by Food and Drug Administration for the treatment of relapsed and refractory MM [23]. HDAC inhibitors in combination with bortezomib (BTZ) have synergistic cytotoxic effects on MM cells by disruption of protein degradation and inhibition of the interaction of MM cells with the tumor microenvironment [24].

Furthermore, alterations in histone methyltransferases can also mediate chemotherapy resistance in MM including cell adhesion-mediated drug resistance (CAM-DR) which is a rather complex and poorly explored form of DR in MM. Kikuchi et al. demonstrated that direct adhesion to bone marrow stromal cells inactivated (phosphorylated) the histone methyltransferase enhancer of zeste homolog 2 (EZH2) which resulted in H3K27 (histone H3-Lysine 27) hypomethylation. This in turn led to sustained expression of anti-apoptotic genes such as IGF1, BCL2, and hypoxia inducible factor 1-α (HIF1A) [25]. The above study identifies stroma-induced histone hypomethylation as a mechanism of CAMDR in MM hence a tumor suppressor function of EZH2. In addition, CDK1-dependent inactivation (phosphorylation) of EZH2 and subsequent H3K27 hypomethylation also leads to resistance to tyrosine kinase inhibitors (TKIs) and cytotoxic drugs in AML [26].

On the other hand, oncogenic functions of EZH2 have also been reported by some studies. For instance, it was shown that silenced polycomb target genes were more frequent in MM and ChIP-seq profiling data revealed increased number of silenced H3K27me3 (Histone H3 lysine 27 trimethylation) target genes in MM patients at advanced stages of the disease, and the expression pattern of H3K27me3-marked genes was correlated with poor patient survival [27, 28]. In addition, pharmacological inhibition of EZH2 reduced the expression of some MM-associated oncogenes [29], it also caused reduction of H3K27me3 level in EZH2 target genes in MM cells promoting the expression of EZH2-repressed tumor suppressor genes and subsequently blocked the cell proliferation and invasion [30, 31]. In addition to EZH2, the histone methyltransferase MMSET/WHSC1, which is overexpressed in MM patients with t(4;14), is known to be a driving factor in the pathogenesis of this MM subtype. Shah et al. showed that MMSET/WHSC1 could enhance DNA damage repair and lead to DR in MM and that depletion of MMSET enhanced the efficacy of chemotherapy, inhibited tumor growth, and extended survival in a mouse xenograft of t(4;14) KMS11 MM cells [32].

It is important to note that most studies concerning epigenetics in MM pathogenesis focused on EZH2-mediated transcription repression of target genes and it is not clear whether somatic mutations causing EZH2 gain or loss of function could also play a role in MMDR. Indeed, both types of mutations have been reported in other hematologic malignancies leading to biologic and clinical outcomes that indicate context-dependent tumor suppressor or oncogenic function of EZH2 [33]. Taken together, epigenetic mechanisms including DNA methylation and histone methylation/deacetylation play an important role in MM pathogenesis particularly DR by regulating expression of target genes with established functions in cell viability and apoptosis.

### A triad of “miRNA-drug-target” shapes the drug response of MM cells

**Fig. 1:**
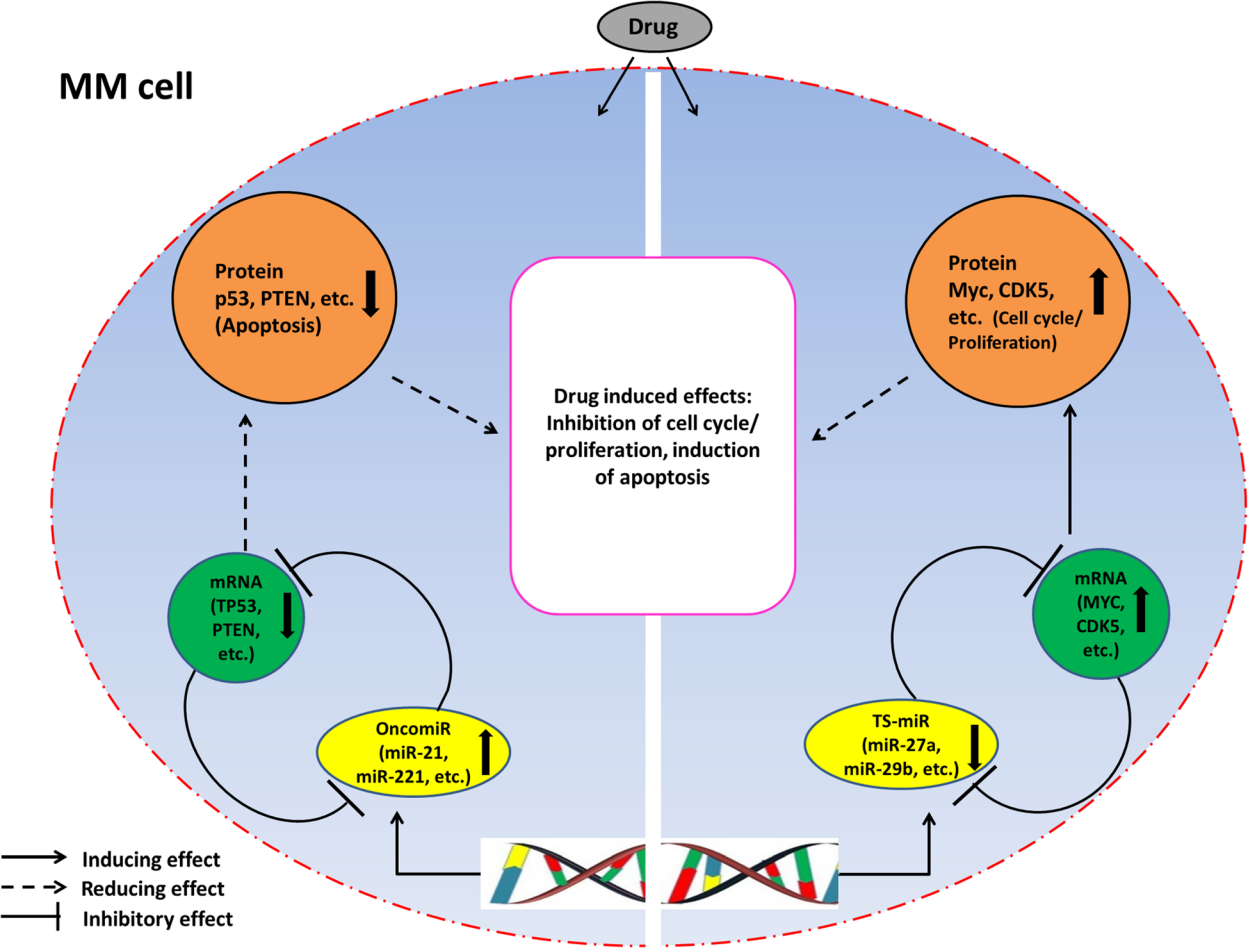
Schematic illustration of miRNA-target-drug axis in MM cells. When MM cells are exposed to the anti-myeloma drugs, through as-yet unclear mechanisms, the drugs may interact with either miRNA processing machinery (genomic or post-transcriptional) or their targets. In the context of TS-miRs (e.g., miR-29b, miR-27a), their oncogenic targets, e.g., MYC or CDK5, will be overexpressed leading to induction of cell proliferation or inhibition of apoptosis (attenuation of drug effect). On the other hand, when the context is dealing with oncomiRs, e.g., miR-21 or miR-221/222, their TS targets (p53, PTEN) will be suppressed culminating in the same outcome as above. It is still unclear whether the expression of two types of miRNAs is in fact governed initially by the oncogenic process or the drug exposure “manipulates” the genome or post-transcriptional system to modulate miRNA expression

Many studies have demonstrated that miRNAs could be involved in DR of MM (listed in Table 1). It has recently been suggested that miRNAs can indirectly affect the efficacy of an anti-tumor drug depending on whether their target has negative or positive impact on the drug function [34]. This concept extends the function of miRNAs beyond what we know as stunning performers in the genome regulating expression of genes and denotes a significant role of these small molecules in DR of tumor cells [15]. However, miRNA expression pattern which would possibly be altered by the neoplastic context is the determining factor. This means downregulated miRNA (TS-miRNA) can boost or lower the efficacy of the drug, respectively, if the protein targeted by a specific miRNA promotes or dampens drug effects. The contrasting scenario will apply when the expression level of miRNAs in tumor context is high (OncomiR). MiR-221/222 and miR-21 are two known oncogenic miRNAs with high expression in MM [35–38] and other cancers [39–42]. They target the tumor suppressor PTEN and pro-apoptotic PUMA, two proteins known to be upregulated by BTZ [43, 44]. In addition, miR-451 regulates stemness of MM side population and inhibition of this miRNA enhances anti-myeloma agents’ effectiveness, through increasing cells apoptosis and reducing MDR1 (multidrug resistance 1) gene expression [45]. These miRNA-target interactions had negative impact on drug function in tumor cells, hence occurrence of DR. Notably, synthetic inhibitors of the oncomiRs miR-21 and miR221/222 have been successfully administered to preclinical models of MM yielding prominent anti-tumor effects [35–38]. MiR-27a was identified as a tumor suppressor to be downregulated in MM [46] and leukemia [47] cells and targeted the oncogenes CDK5 and P-glycoprotein, respectively, which were highly expressed in tumor cells culminating in the same outcome as above. MiR-29b is another example of TS-miRNAs which was significantly reduced in BTZ-resistant cells as well as in cells resistant to second-generation PIs carfilzomib and ixazomib. miR-29b targeted the proteasome activator PA20 and disrupted aggresome/autophagosome formation to enhance the anti-myeloma effects of BTZ [48]. It is not surprising to expect that the target of the miRNA in this triad could also be an epigenetic modifying enzyme like EZH2 or HDACs whereby their interaction would possibly function through an established loop to sustain MM cell drug response (see below for further explanations). These statements highlight the notion that the function of an anti-myeloma drug, e.g., BTZ, or how the MM cells respond to the drug can be shaped by the pattern of miRNA-target interaction which in some cases will end in therapy resistance. The above scenario has been illustrated in Fig. 1.

### Epigenetic modifications and miRNAs interplay: a scenario in the context of anti-myeloma drug

MiRNAs have a large impact on tumorigenesis by modulating expression of various oncogenes or tumor suppressors and also contribute to DR. Hence identifying the regulatory mechanisms of miRNA expression will be very helpful to understand underlying mechanisms of DR in MM and to find more effective therapeutic targets. Recent epigenetic investigations have identified promoter hypermethylation of tumor suppressor miRNAs (TS-miRNAs) in many cancer types including MM [11]. MiR-34a/b/c, miR-124-1, miR-194-2-192, miR-203, miR-152, and miR-10b-5p in MM were reported to be silenced by DNA hypermethylation [11, 49, 50]. Importantly, most target genes of these miRNAs encode for proteins involved in survival, proliferation, and DR. For instance, hypermethylation-mediated inactivation of miR-34a/b/c attenuates tumor suppressor activity of p53, as these miRNAs are known to be direct transcriptional targets and tumor-suppressive effectors downstream to p53. This in turn will lead to loss of translational repression of miR-34a/b/c targets, BCL-2, CCND1, CCNE2, CDK4, CDK6, E2F, and v-MYC.

Studies from our group [51, 52] and others [53] have identified miR-137 as a TS-miRNA whose overexpression in MM cells sensitizes them to anti-myeloma drugs. MiR-137 in MM was silenced by promoter hypermethylation which was associated with chromosomal instability (CIN) and resistance to BTZ in MM cells. AURKA, a gene coding for proteins involved in mitosis and cell proliferation, was identified as a direct target of miR-137. Ectopic expression of miR-137 sensitized the cells to BTZ by upregulating p53 and downregulating ATM/Chk2 indicating that epigenetically regulated miR-137 plays role in DR of MM cells by maintaining a proliferation or survival pathway [52].

Moreover, some oncogenes are targeted by hypermethylated miRNAs in MM and hypomethylation of miRNA genes by using DNMT inhibitors can downregulate those oncogenes and inhibit cell growth and induce apoptosis in MM cells. For example, RecQ helicases (DNA unwinding enzymes involved in the maintenance of chromosome stability) are significantly upregulated in MM and protect MM cells from melphalan and bortezomib cytotoxicity. DNMT inhibitor treatment of MM cells results in RECQ1 downregulation through miR-203 demethylation and sensitizes cells to anti-tumor drugs suggesting that epigenetic modifier could be useful for treatment of relapsed cases [54].

Resistance to drugs could also be associated with histone modifications (deacetylation, methylation) of miRNA promoters, another epigenetic mechanism regulating expression of miRNAs in cancers [14, 55, 56], although far less investigated in MM. For instance, the HDACs 1, 2, 3, and 4, DNMTs, acetylated H2B, and acetylated H3 were direct targets of several miRNAs in doxorubicin-resistant lung cancer cell lines and were in fact in a functional interaction with these miRNAs [14]. The histone methyl transferase MMSET is overexpressed in about 15% of MM patients due to the t(4;14) translocation. MMSET overexpression induced c-MYC expression in MM cells by repressing miR-126* which targeted c-MYC, hence increase in proliferation of MM cells. It was shown that MMSET bound to miR-126* promoter which was indicated by increased H3K9 trimethylation and decreased H3 acetylation, leading to miR-126* repression [57]. Although drug response of MM cells was not explored in this setting, it may be speculated that resistance to drug could also happen due to c-MYC-mediated increased proliferation of MM cells. In support of this, cell cycle-mediated drug resistance has been suggested as a critical phenomenon impeding combined chemotherapies, which warrants development and incorporation of cell-cycle inhibitors [58].

**Fig. 2:**
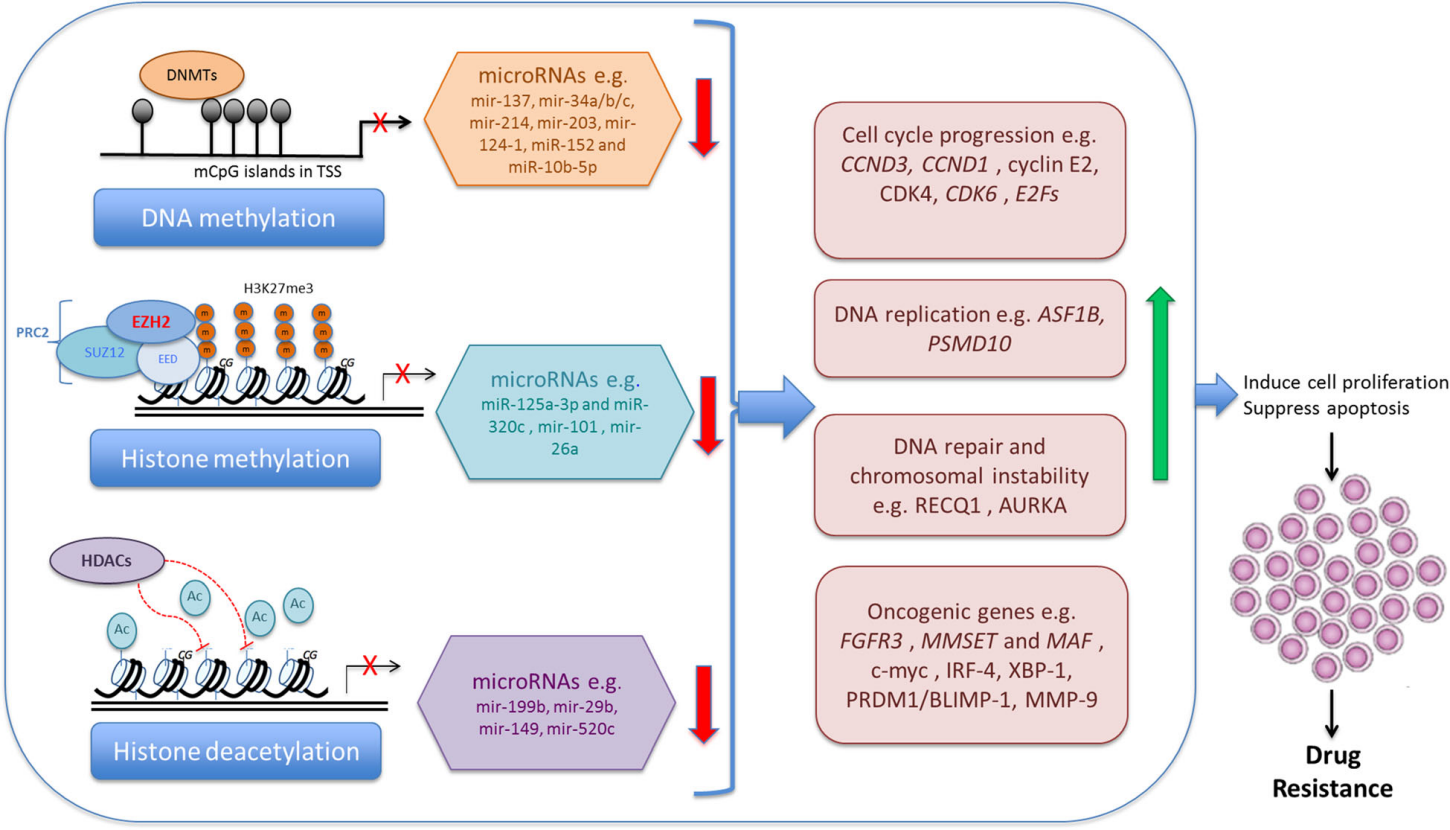
Schematic illustration of miRNA regulation by epigenetic modifiers in MM. Epigenetic modifications such as DNA methylation, histone methylation and histone deacetylation suppress tumor suppressor miRNAs. The miRNAs target downstream genes that are involved in different cell processes such as cell proliferation, apoptosis and DNA repair. Suppression of miRNAs by epigenetic dysregulation leads to overexpression of these target genes which determine the phenotype of DR in MM

The chromatin remodeling enzyme EZH2 is probably the most attractive epigenetic modifier in cancers [33, 59, 60] which has been shown to induce DR in tumor cells by silencing miRNAs and establishing a functional mutual interaction with miRNAs [13]. EZH2 has also been shown to interact with transcription factors which are targets of tumor suppressor miRNAs, such as MYC in lymphomas [61] and in MM [29]. Alzrigat et al. demonstrated that pharmacologic inhibition of EZH2 in MM cell lines and primary cells suppressed transcription factors with oncogenic activity in MM including IRF-4, XBP-1, PRDM1/BLIMP-1, and c-MYC. In parallel, EZH2 inhibition reactivated the expression of TS-miRNAs, miR-125a-3p, and miR-320c, which were also targets of EZH2 and H3K27m3 [29]. Additionally, miR-138 that targets EZH2 is suppressed in drug-resistant phenotypes of MM cells, restoration of this miRNA using EZH2 silencing or pharmacologic inhibition reverses DR and sensitizes MM cells to drug-induced toxic effects (our unpublished data). These observations provide evidence that the epigenetic modifier EZH2 contributes significantly to MM cell proliferation and DR by targeting TS-miRNAs. Figure 2 illustrates the miRNA/epigenetic modifier enzyme interactions which are involved in MMDR.

It is interesting to note that a group of miRNAs, termed “epi-miRNAs”, has been reported to reciprocally modulate epigenetic regulators, suggesting the existence of a regulatory circuit between miRNAs and epigenetic modifiers [62]. The best example in MM is miR-29b which was shown to specifically target HDAC4 in a mutually functional loop [63, 64]. Silencing/inhibition of HDAC4 triggered apoptosis, enhanced drug (bortezomib and dexamethasone)-induced cell death, and upregulated miR-29b in MM cells (by promoter hyperacetylation). On the contrary, overexpression or inhibition of miR-29b, respectively, antagonized or potentiated the anti-myeloma effects of the pan-HDAC inhibitor SAHA confirming that HDAC4-miR-29b axis modulates the effects of anti-myeloma drugs. The key epigenetic modifiers found in MM and their miRNA targets are summarized in Table 2.

**Table 2:** Summary of key epigenetic modifiers found in MM and their miRNA targets

Sample source	Epigenetic modifiers	miRNAs targeted	Targets/pathways modulated by miRNA	Functional outcomes	Refs.
MM cell lines (NCI-H929, U266, KMS11, OPM2, RPMI8226, MM1.S) and MM primary samples	DNA methylation (DNMTs)	miR-137	AURKA/p-ATM, p-Chk2	Induction of drug resistance to bortezomib and epirubicin, chromosomal instability	[52]
MM cell lines (MM1S, H929, OPM-2, JJN3, and RPMI 2886)	DNA methylation (DNMTs)	miR-214	PSMD10 & ASF1B/p53-MDM2	A significant enrichment for DNA replication and induction of cell proliferation, and as a consequence also in cell survival	[87]
MM cell lines (NCI-H929 and U-266, KMS-12-PE, LP-1, OPM-2) and MM primary samples	DNA methylation (DNMTs)	miR-124-1	CDK6	Induction of cell proliferation	[50]
MM cell lines (KMS11, SKMM1, and NCI-H929) and PCL and MM primary samples	HDACs	miR-29b	Mcl1/SP1 and HDAC4	Induction of cell growth by upregulation of pro-survival proteins (MCL-1 and SP1)	[63]
MM cell lines (MM.1S, LP1, H929, and JJN3)	HDACs	miR-9-5p	IGF2BP3/CD44	CD44 overexpression, a glycoprotein that has been associated with lenalidomide and dexamethasone resistance in myeloma	[90]
MM cell lines (INA-6, LP-1, L363, KMS-11) and MM primary samples	EZH2	miR-125a-3p & miR-320c	RF-4, XBP-1, BLIMP-1, c-MYC	Upregulation of oncogenes and inhibit apoptosis	[29]
MM cell lines RPMI8226 and U266	EZH2	miR-101	E-cadherin, MMP9, c-Myc, cyclin D3, CDK4, and CDK6	Induction of cell proliferation and inhibit apoptosis	[91]

Taken all together, in the setting of MM cells, the mutual interaction between miRNAs and epigenetic markers plays an important role in regulation of drug response of MM cells.

### Clinical application of epigenetic inhibitors in combination with miRNAs in MM

Strategies for clinical application of epigenetic inhibitors including DNMT, HDAC, and HAT inhibitors in MM therapy have been reviewed elsewhere [65, 66]. Generally, these inhibitors have been administered in combination regimens in MM. For instance, EZH2 inhibitors have been applied to clinical trials in lymphoma and are suggested as promising therapeutic strategy in MM in combination with IMiDs [8] and proteasome inhibitors [67]. Kikuchi et al. showed that HDACs were critical targets of BTZ and knockdown of HDAC1 enhanced BTZ-induced apoptosis, whereas HDAC1 overexpression conferred resistance to BTZ in MM cells, suggesting that combination of BTZ and HDAC inhibitors could be a more efficient treatment strategy for MM [68]. Indeed, HDAC inhibitors have also been applied to clinical therapies of MM in combination with IMiDs or proteasome inhibitors [69, 70]. While miRNA mimics have been tested in many pre-clinical studies in MM, obstacles to apply these agents to MM clinical trials still persist [71]. Efficient delivery of nucleic acids into tumor tissues and their uptake specifically by the tumor cells have been stressed to be the challenging issues. On the other hand, considering an established functional interaction between miRNAs and epigenetic regulators, which regulates MM cell drug responses, combination of miRNA mimics with inhibitors of these modifiers could be a more potent therapeutic strategy in MM patients in relapse or refractory to treatments.

## Conclusions

Current era of MM therapy is witnessing the significant progress of strategies and approaches aiming mostly at overcoming the DR. While most novel treatments including proteasome inhibitors especially in combination modalities have proved to increase the survival of patients, MM still remains to be drug resistant and most patients relapse or become refractory. Studies have demonstrated that miRNA may be applied for the targeted delivery of personalized medicine to improve the outcome of MM patients [72]. Furthermore, the number of studies focusing on pre-clinical applications of miRNAs in MM is increasing; however, concerns and obstacles to these approaches in terms of translation to clinic still persist [71]. Epigenetics is perhaps taking an exciting and promising position at the frontier of MMDR mechanisms. Taking advantage of epigenetic regulation of miRNAs, future studies should attempt to examine therapeutic application of epigenetic markers to restore TS-miRNAs in MM pre-clinical models. This strategy will especially be promising when MM cases with resistance to HDAC inhibitors are dealt with [73].

In conclusion, epigenetics-miRNA axis plays a crucial role in MM pathogenesis and could provide potential therapeutic targets. However; due to limited studies, further in-depth studies in this regard are required to open a novel and exiting venue to understand the underlying mechanism of DR in MM, which tends to be the outstanding obstacle to MM therapy.

## Abbreviations

AML: Acute myeloid leukemia
AURKA: Aurora kinase A
BCL-2: B cell lymphoma 2
BTZ: Bortezomib
CAM-DR: Cell adhesion-mediated drug resistance
CCND1: Cyclin D1
CCNE2: Cyclin E2
CDK4,5,6: Cyclin-dependent kinase 4,5,6
CDKN2A,B: Cyclin-dependent kinase inhibitor 2 A,B
ChIP-seq: Chromatin immunoprecipitation followed by sequencing
CIN: Chromosomal instability
DNMT: DNA methyl transferase
DR: Drug resistance
EZH2: Enhancer of zeste homolog 2
HAT: Histone acetyl transferase
HDAC: Histone deacetylase
HIF1A: Hypoxia inducible factor 1-α
IFR4: Interferon regulatory factor 4
IGF1: Insulin growth factor 1
IMiDs: Immunomodulatory drugs
MDR1: Multidrug resistance 1
MM: Multiple myeloma
MMDR: Multiple myeloma drug resistance
MMSET/WHSC1: Multiple myeloma SET domain/Wolf-Hirschhorn syndrome candidate 1
PCL: Plasma cell leukemia
PI: Proteasome inhibitor
PRDM1/BLIMP1: PR domain zinc finger protein 1/B lymphocyte inducer of maturation program 1
PTEN: Phosphatase and tensin homolog
PUMA: P53 upregulated modulator of apoptosis
TKI: Tyrosine kinase inhibitor
TS-miRNA: Tumor suppressor micro-RNA
